# The Sociocultural Determinants of Cervical Cancer Outcomes in India: A Critical Review of Diagnostic Delays and Treatment Disparities

**DOI:** 10.7759/cureus.88967

**Published:** 2025-07-29

**Authors:** Santhosha Kulal, Jayashree H K, Arpita Shivapura Anandakumar, Rashmi H R, Pushpa G

**Affiliations:** 1 Radiation Oncology, Sri Devaraj Urs Academy of Higher Education and Research, Kolar, IND; 2 Pathology, Sri Chamundeshwari Medical College Hospital & Research Institute, Mandya, IND; 3 Anaesthesiology, Sri Chamundeshwari Medical College Hospital & Research Institute, Mandya, IND; 4 Obstetrics and Gynaecology, Jagadguru Sri Shivarathreeshwara (JSS) Medical College, Mysore, IND

**Keywords:** barriers to cervical cancer screening, cervical cancer in india, disparities in cancer care, hpv vaccination and screening healthcare, sociocultural barriers to cancer prevention

## Abstract

Cervical cancer remains a major health concern in India, despite being preventable through human papillomavirus (HPV) vaccination and routine screening. This review examines the key barriers that prevent early detection and treatment, focusing on healthcare system challenges, sociocultural influences, and economic factors. The findings highlight that many women, especially those from rural and lower-income communities, do not have access to proper screening or vaccination due to a lack of awareness, financial difficulties, and inadequate healthcare facilities. Cultural stigma and misinformation about HPV vaccines further discourage preventive measures.

The healthcare system itself faces limitations, including a shortage of trained medical professionals, insufficient screening programs, and delays in diagnosis. Many women are only diagnosed when the disease has already progressed, making treatment less effective. Regional differences also play a role, with some states having better access to care while others struggle with limited resources.

To reduce the impact of cervical cancer in India, this review suggests increasing awareness through community-based education programs, integrating HPV vaccines into the national immunization plan, and improving access to screening facilities, especially in rural areas. Training healthcare workers to educate and encourage women to get screened and vaccinated can also lead to better outcomes. Mobile screening units and digital health initiatives can help further reach underserved populations.

Future research should focus on evaluating the effectiveness of these interventions, understanding the reasons behind vaccine hesitancy, and exploring new ways to improve healthcare access for women. A coordinated approach involving the government, healthcare providers, and community organizations is necessary to ensure that all women, regardless of their background, have access to life-saving prevention and treatment options.

## Introduction and background

Cervical cancer remains one of the major public health challenges for women worldwide, with disproportionate morbidity and mortality rates in low and middle-income countries (LMICs) [1]. Despite being largely preventable through early screening, timely diagnosis, and vaccination against the human papillomavirus (HPV), India continues to bear a significant burden of cervical cancer cases and deaths. Cervical cancer is the second most common cancer among Indian women, accounting for nearly 22% of all cancer-related deaths, falling behind breast cancer [2]. India alone contributes to nearly one-third of global cervical cancer deaths, with women facing a 1.6% cumulative risk of developing cervical cancer and a 1% cumulative risk of mortality from the disease [2].

Unlike high-income nations, where organized screening programs and widespread HPV vaccination have significantly reduced cervical cancer incidence, India faces challenges in screening uptake and vaccination coverage [3,4]. While the Government of India has made screening available for common cancers under national programs, data from the National Family Health Survey (NFHS-5) indicate that screening rates among women aged 30-49 years were alarmingly low [5]. Studies report that only 1.9% of Indian women have ever undergone cervical cancer screening, with significantly lower participation rates among rural and low-income populations [6]. Furthermore, a comparative analysis of NFHS-4 and NFHS-5 data reveals a sharp decline in the proportion of women reporting cervical examinations across all age groups and education categories [7].

Although India has seen a decline in cervical cancer incidence and mortality rates over the past three decades, with states such as Jharkhand and Himachal Pradesh recording the highest percentage decrease in cervical cancer incidence and mortality [8]. Northeastern states, such as Mizoram, Arunachal Pradesh, and Nagaland, continue to experience a high burden of cervical cancer, with disability-adjusted life years (DALYs) exceeding 300 per 100,000 women [9]. Districts with lower human development indices, such as Aizawl, Dibrugarh, and Kollam, also report significantly higher age-truncated incidence rates [10].

The availability and utilization of cervical cancer screening programs, such as those implemented under the National Health Mission, have contributed to the decline in incidence in some regions. However, screening coverage remains low in many states, with significant disparities between urban and rural areas [11]. For example, while Tamil Nadu reports screening rates as high as 10.1%, states like Assam and West Bengal have rates as low as 0.2%, highlighting inequities in healthcare infrastructure and access [12].

Despite the overall decline in incidence, the burden of cervical cancer in India is projected to rise, with an estimated 1.5 million DALYs by 2025, underscoring the urgency for enhanced prevention and control measures [9]. The availability of preventive measures such as HPV vaccination and cervical cancer screening in India continues to face significant disparities in early detection and treatment, particularly among marginalized and low-income populations [13]. The persistent gaps in screening uptake, regional disparities in cervical cancer incidence, and the projected rise in DALYs indicate that current interventions are insufficient to address the full scope of the problem. Existing literature primarily focuses on epidemiological trends, while comprehensive analyses of systemic barriers ranging from healthcare infrastructure to sociocultural and provider-related challenges remain limited.

Due to the urgency of addressing cervical cancer disparities in India, this review synthesizes the multi-faceted barriers contributing to delayed diagnosis, treatment inequities, and systemic gaps and disparities, highlighting the need for targeted interventions, improved healthcare accessibility, and culturally sensitive awareness programs to enhance prevention, early detection, and treatment outcomes. This paper provides a comprehensive analysis of cervical cancer in India, examining its epidemiological trends and the structural challenges within the healthcare system. It further explores the sociocultural determinants influencing screening uptake, diagnostic delays, and treatment disparities. Finally, the review discusses policy interventions and strategic recommendations to improve early detection, equitable treatment, and overall cervical cancer outcomes in India.

## Review

Healthcare system barriers and inefficiencies

Deficiencies in Healthcare Infrastructure

According to research, a large majority of women in India, around 86%, report never having undergone cervical cancer screening, highlighting a significant gap in preventative healthcare practices related to this disease in the country [14]. There is a marked inequality in cervical cancer screening, with higher prevalence among wealthier populations in regions like the Northeast and South, while the central region of India has a low prevalence [15]. The prevalence of cervical cancer screening is notably low in these regions, with West Bengal having a screening prevalence of only 0.2% [15]. Urban women (2.4%) are marginally more likely to be screened compared to their rural counterparts (1.8%) [16]. However, even within urban centers, women from low-income neighborhoods, including slums, encounter significant barriers to screening, as they frequently rely on overburdened and underfunded public healthcare facilities.

The consequences of these low screening rates are severe, as they lead to delayed diagnoses, with 67.2% of cervical cancer cases detected only after the disease has metastasized beyond the cervix [11]. Many women seek medical intervention only after the disease has reached an advanced stage, which reduces the likelihood of successful treatment and increases mortality rates, as the survival rates drop significantly for patients diagnosed at a later stage [17]. A study reported a three-year survival rate of only 45% for women with late-stage cervical cancer [18]. In another study conducted in South India, the five-year overall survival rate for patients with advanced stages (Stage III & IV) was significantly lower, with a hazard ratio indicating a threefold increase in the risk of death compared to early-stage patients [19]. Patients diagnosed with early-stage cervical cancer have a significantly higher survival rate [18].

Healthcare infrastructure limitations and resource constraints play a significant role in delayed cervical cancer diagnoses across India. Access to healthcare services, including screening and vaccination, varies significantly across regions, affecting incidence rates [11]. Despite national guidelines recommending routine screening, many primary health centers (PHCs) lack the necessary infrastructure to conduct effective cervical cancer screenings [20]. This challenge is particularly prominent in rural regions, where healthcare facilities are scarce, further exacerbating the gap in screening accessibility. Urban areas are equipped with specialized cancer treatment facilities, including advanced screening and therapeutic options, whereas rural regions often lack adequate medical resources, resulting in delayed diagnosis and limited treatment access [21]. Rural areas suffer from severe shortages of healthcare infrastructure and trained medical professionals, resulting in significantly lower screening rates [22,23]. The COVID-19 pandemic further widened these disparities, as rural communities faced additional barriers such as increased poverty levels and reduced outreach efforts [23].

Furthermore, the inadequacy of India’s healthcare infrastructure in supporting nationwide cervical cancer screening and treatment is a key challenge. The absence of a national, organized screening program and limited availability of Pap smear testing further compound the issue, particularly among uneducated and underserved populations [24,25]. The lack of a systematic screening initiative and the shortage of trained medical personnel contribute to persistently low screening rates [26]. Several screening techniques, including visual inspection with acetic acid, visual inspection with Lugol’s iodine, Pap smear, and HPV-DNA testing, have been proposed and tested in low-resource settings [27]. Among these, cervical cytology screening has demonstrated effectiveness in reducing disease incidence [27]. However, its implementation remains challenging due to logistical constraints, such as a shortage of trained healthcare personnel and inadequate outreach efforts [28].

Addressing these limitations necessitates infrastructure improvements and enhanced workforce training to facilitate greater screening accessibility. A pilot project in Haryana demonstrated the critical need for healthcare worker training and logistical support to improve screening uptake [20]. Although initiatives such as Ayushman Bharat and the National Cancer Grid aim to expand healthcare access and promote awareness, their effectiveness is largely dependent on their ability to reach and educate the target population [13]. Community-driven efforts, such as the Cancer Awareness, Prevention and Early Detection Trust (CAPED), have demonstrated success in increasing screening rates through grassroots outreach and governmental collaboration [20]. Similarly, the National Programme for Prevention and Control of Cancer, Diabetes, Cardiovascular Diseases and Stroke (NPCDCS) includes cervical cancer screening, yet its implementation remains inconsistent across different regions, limiting its impact [11]. Without comprehensive national policies that prioritize organized screening programs, greater accessibility to testing facilities, and culturally sensitive awareness campaigns, the burden of cervical cancer in India will persist.

Healthcare Provider Biases and Systemic Challenges

The role of healthcare providers in facilitating early cervical cancer detection remains suboptimal. A considerable proportion of healthcare professionals fail to educate and encourage women to undergo screening, particularly during asymptomatic stages, which is a crucial window for early detection. Studies reveal that only 22.16% of women who interacted with healthcare providers were advised to undergo cervical cancer screening, and an even lower proportion (15.18%) underwent testing, despite having access to healthcare facilities [29].

Furthermore, insufficient training and awareness among healthcare professionals significantly limit the reach and effectiveness of screening programs. Many healthcare workers, especially in rural areas, lack the necessary knowledge and resources to properly educate and screen women for cervical cancer [30]. Even physicians themselves have acknowledged the need for additional training and institutional support to implement effective cervical cancer screening and prevention services [30].

Cultural misconceptions further compound these issues, influencing healthcare providers’ hesitancy in recommending screening. Prevailing beliefs linking cervical cancer screening to sexual activity rather than preventive healthcare delay early detection efforts, particularly in socially conservative communities [31]. Additionally, misconceptions regarding the HPV vaccine’s safety and efficacy further hinder its recommendation by healthcare professionals, limiting one of the most effective interventions against cervical cancer [26]. These barriers illustrate not only individual provider biases but also systemic inadequacies in medical training and public health education.

The lack of trained professionals, fragmented healthcare pathways, and inadequate budget allocations significantly restrict access to preventive care [13]. Many women experience prolonged diagnostic delays, requiring multiple visits to different providers before obtaining a definitive diagnosis. Studies report a median diagnostic delay of 66 days, underscoring the inefficiencies and structural weaknesses of the healthcare system [32]. These prolonged delays not only increase disease progression risks but also diminish the effectiveness of available treatment options, ultimately contributing to higher mortality rates.

The role of nurses and community health workers is particularly crucial in bridging awareness gaps and facilitating access to screening and vaccination services. Their active involvement in community-based education initiatives has been shown to increase screening uptake and improve health-seeking behaviors among women, particularly in underserved regions [33]. However, the impact of these initiatives remains limited due to resource constraints and inadequate institutional support [34].

Sociocultural determinants of cervical cancer in India

Lack of Awareness and Education

A major barrier to HPV vaccine acceptance in India is the widespread lack of awareness about cervical cancer and its prevention. Studies show that both marginalized and empowered young women have limited knowledge about HPV and its vaccine, exacerbated by inadequate media outreach [35,36]. A North India study found that only 28.5% of women were aware of cervical cancer, while 71% were unaware of its curability if detected early [37]. In Bihar, only 2.63% of adolescent girls knew about the HPV vaccine, and 12.94% had heard of cervical cancer [38]. Even among young adult female students in Bhopal, awareness regarding cervical cancer as a vaccine-preventable disease remained limited, underscoring the pervasiveness of misinformation and a lack of targeted health education efforts [39].

The urban-rural divide in awareness is particularly pronounced. In Haryana, urban women demonstrated significantly higher levels of knowledge about cervical cancer compared to their rural counterparts [40]. While in rural Maharashtra, despite 64% of women expressing positive attitudes toward screening, only 17% possessed adequate knowledge of the disease [41]. Similar trends were observed in the urban slums of Kolkata, where a lack of knowledge about risk factors and symptoms leads to delayed diagnosis and poorer outcomes [42].

In Davanagere, Karnataka, none of the surveyed women who were attending the Obstetrics and Gynecology Outpatient Department (OBG OPD) at a hospital had received the HPV vaccine, and awareness regarding its preventive role was minimal [43]. Beyond the general population, knowledge deficiencies are also evident among healthcare students and professionals. Even among medical students, a significant proportion lacked awareness of the vaccine’s importance. 24% did not know about cervical cancer screening, 32% had never heard of Pap smear, and 11% had never heard of the HPV vaccine, pointing to the systemic gaps in professional health education that could otherwise facilitate advocacy and improve vaccination uptake [44].

A critical determinant of awareness and preventive healthcare participation is education. Women with higher levels of education are demonstrably more likely to be informed about cervical cancer and participate in screening programs, while those with limited or no formal education remain significantly disadvantaged [45]. This educational disparity has a direct impact on screening rates, which remain critically low across India. National data indicates less than 2% of women undergo cervical cancer screening, reflecting the strong correlation between inadequate awareness and poor engagement in preventive healthcare measures [45]. Women with no formal education and higher parity (four or more pregnancies) are at significantly greater risk of developing cervical cancer [28]. These challenges substantially undermine the effectiveness of vaccination programs aimed at reducing cervical cancer incidence.

Socioeconomic Barriers to Prevention and Treatment

Socioeconomic factors play a pivotal role in shaping access to cervical cancer screening and treatment in India. One of the most striking manifestations of socioeconomic inequality is the wealth-based disparity in cervical cancer screening. Women from higher incomes exhibit a substantially higher screening participation rate and can access such services [15].

Multiple structural barriers are responsible for hindering the participation of lower socioeconomic status (SES) women in both cervical cancer screening and treatment. Limited awareness, cultural stigmas, economic constraints, and inadequate healthcare infrastructure disproportionately affect rural communities, further reducing their engagement with preventive healthcare services [46]. Women from lower-income backgrounds and those with limited education face more barriers to healthcare access, including financial constraints, lack of awareness, and inadequate healthcare facilities [24].

For many, especially in rural areas, the economic burden makes early screening and treatment unfeasible [47]. Moreover, financial constraints extend beyond direct medical costs to include indirect barriers such as transportation expenses, opportunity costs associated with medical visits, and the potential loss of daily wages. Women in rural India struggle to travel long distances to healthcare facilities, preventing them from attending regular screenings or seeking timely medical intervention [21]. These logistical challenges, combined with financial constraints, lead to poorer health outcomes and higher late-stage cervical cancer diagnoses.

Women with higher educational attainment, access to government health insurance, and greater financial stability are far more likely to participate in screening programs, illustrating the profound influence of economic security and awareness on preventive healthcare utilization [15,45].

Cultural and Social Stigma

Sociocultural factors, including gender norms, stigma, and societal expectations, significantly hinder women’s ability to seek timely medical intervention for cervical cancer in India. Women often prioritize domestic responsibilities over their health, leading to delays in screening and treatment [37]. Gender roles and patriarchal societal structures further compound these challenges, as women in conservative households often require spousal or familial permission to access healthcare [41]. Dependence on male family members for financial and logistical support not only restricts women’s autonomy but also reduces the likelihood of seeking preventive care [16].

Despite strong evidence supporting the efficacy of the HPV vaccine, misinformation and religious concerns have contributed to its low uptake [13]. In India, there is a perception that the vaccine is associated with sexual activity, which is a sensitive topic for many [36]. Additionally, fear of social stigma and embarrassment surrounding gynecological health prevents many from participating in screening programs, with studies indicating that a considerable number of women refrain from seeking treatment due to these concerns [47].

Women from conservative Muslim and Hindu sects often face additional barriers due to social norms that discourage conversations about reproductive health [16]. Cultural and religious taboos surrounding reproductive health further restrict open discussions, discouraging women from accessing screening or treatment [13,37]. Even among women who are aware of cervical cancer, pervasive fatalistic beliefs and misconceptions deter them from taking preventive action. Many perceive a cancer diagnosis as a death sentence, reinforcing fear and discouraging early detection efforts [41].

Public mistrust of vaccines, coupled with misinformation and past controversies, has led to skepticism and resistance, significantly lowering vaccination rates across India [13]. Fear, fatalism, and the low prioritization of self-care further compound these barriers, making it difficult for women to access necessary healthcare services [37].

Discussion

Cervical cancer remains a critical public health challenge in India despite advancements in vaccination and screening initiatives. This review highlights that systemic barriers such as vaccine hesitancy, inadequate healthcare infrastructure, shortage of trained professionals, and socioeconomic disparities hinder effective prevention and early detection efforts. Without targeted interventions, low screening rates, delayed diagnoses, and poor cervical cancer outcomes will persist, disproportionately affecting marginalized women in India.

To address these issues successfully, Governments, NGOs, and healthcare providers must collaborate to develop sustainable, community-based interventions. The success of HPV vaccination programs in countries like Belgium and Australia can serve as a model for India [13]. Moreover, strong policy implementation and monitoring mechanisms are required to track progress and ensure accountability in achieving cervical cancer elimination targets.

Although programs like Ayushman Bharat seek to provide universal health coverage, their influence on HPV vaccination uptake remains limited. Incorporating the HPV vaccine into India’s Universal Immunization Program (UIP) could significantly improve its affordability and accessibility, particularly among disadvantaged populations [13]. The introduction of CERVAVAC, a domestically produced and cost-effective HPV vaccine, presents an opportunity to enhance vaccination efforts and should be widely deployed. Furthermore, leveraging the digital infrastructure under the National Health Mission (NHM) could facilitate widespread cervical cancer screening and vaccination awareness [13].

Mandatory HPV vaccination for girls aged 9-14 in public schools and subsidized options for private institutions can significantly improve vaccine coverage [48]. Innovative delivery models, such as single-dose HPV vaccination schedules, which are equally effective as multi-dose schedules, may also enhance compliance rates [13]. Providing parents with digital resources to understand the importance of HPV vaccination could further help address vaccine hesitancy and alleviate concerns [34].

Public awareness campaigns that consider cultural sensitivities are necessary to counter misinformation and improve vaccine acceptance. Developing culturally relevant public health initiatives can help normalize cervical cancer screening and vaccination [48]. NGOs and grassroots organizations should implement door-to-door outreach programs to effectively engage underserved communities. Also, collaboration with community leaders, religious figures, and social influencers could play a crucial role in disseminating accurate information and increasing awareness. Strengthening women's financial and decision-making autonomy by incorporating cervical cancer screening into maternal and reproductive health services could further enhance participation [11].

Addressing cultural stigma through community-driven strategies could improve screening uptake, particularly among vulnerable populations such as tribal communities, urban slum residents, and rural women [49,50]. Enhancing healthcare infrastructure, especially at PHCs, should be a priority to ensure the availability of essential screening equipment, adequately trained medical personnel, and diagnostic facilities [13]. Training healthcare providers and school staff to advocate for HPV vaccination could improve parental trust and acceptance [34]. The CAPED project has demonstrated that training community health workers to facilitate screening services can significantly increase participation, particularly in underserved areas [20]. Regular training programs aimed at improving the ability of gynecologists, pediatricians, and frontline healthcare workers to promote cervical cancer prevention services are needed [34].

Enhancing the screening methods and moving from conventional Pap smear cytology to HPV-based screening can improve early detection rates [49]. To improve accessibility, self-sampling HPV tests and point-of-care screening should be introduced, particularly in rural and low-resource settings [50]. Mobile screening units and integrating HPV testing into routine gynecological check-ups at PHC can further boost the participation of females.

Large-scale awareness programs, such as the “Smart HPV-Immunization and Awareness Programme”, which utilizes digital media, mobile health technology, and artificial intelligence (AI) to promote HPV vaccination, could be instrumental in educating women, families, and communities about cervical cancer prevention [13]. Strategies including mobile health applications, social media engagement, and interactive digital platforms can enhance outreach efforts.

Future research should focus on evaluating the effectiveness of policy-driven interventions aimed at increasing screening coverage and HPV vaccination uptake across diverse socioeconomic and geographic populations. Studies exploring the role of digital health technologies in improving awareness and accessibility could provide innovative solutions for overcoming logistical and infrastructural challenges. Investigating culturally tailored communication strategies to address vaccine hesitancy and stigma, as well as assessing the impact of gender dynamics on healthcare-seeking behaviors, would further contribute to the development of equitable and sustainable cervical cancer prevention programs.

## Conclusions

Despite advancements in preventive measures, systemic barriers continue to hinder early diagnosis and equitable treatment of cervical cancer in India. By adopting a comprehensive, evidence-based strategy, India can overcome the systemic barriers identified in this review and move toward equitable cervical cancer prevention, early detection, and treatment outcomes. These reforms will be instrumental in reducing the burden of cervical cancer and improving health equity for women across the country.
